# Chemoselectivity in the Dehydrocoupling Synthesis of Higher Molecular Weight Polysilanes

**DOI:** 10.3390/ma3021125

**Published:** 2010-02-10

**Authors:** Florian Lunzer, Christoph Marschner

**Affiliations:** Institut für Anorganische Chemie, Technische Universität Graz, Stremayrgasse 16, A-8010 Graz, Austria; E-Mail: florian.lunzer@cytec.com (F.L.)

**Keywords:** polysilane, catalysis, group 4 metallocene, dehydrocoupling polymerization

## Abstract

The Cp_2_ZrCl_2_/2 BuLi catalyzed co-polymerization of H_2_MeSiSiMeH_2_ and PhSiH_3_ was compared to the homo-polymerization of H_2_MeSiSiPhH_2_. In contrast to the co-polymerization, which gave molecular weights comparable to homo-polymerization of phenylsilane, the reaction of 1-methyl-2-phenyldisilane yielded a partially cross-linked high molecular weight polymer with very broad molecular weight distribution. A higher reactivity of phenyl-substituted silicon atoms compared to methyl-substituted ones was detected. Stoichiometric reactions of some disilanes with the slow dehydropolymerization catalyst CpCp*Hf(Cl)Si(SiMe_3_)_3_ gave metal disilanyl intermediates with selectivities that reflect the observed polymerization behavior.

## 1. Introduction

Polysilanes, *i.e.,* long chains of catenated silicon atoms, have attracted considerable attention. The reasons for this interest are strongly linked to the phenomenon of σ-bond conjugation [1,2,3,4]. This unusual property makes polysilanes interesting materials in the fields of optoelectronics, nonlinear optics and microlithography [5].

However, the main process that is used so far for the synthesis of polysilanes is the Wurtz type coupling of dihalogensilanes with alkali metals. Due to the nature of this reaction, the choice of starting materials is restricted to compounds with substituents that can survive these harsh conditions. In addition, stereochemical control of the polymerization is hard to achieve. On the other hand, this process allows one to obtain high molecular weight polymers. Nevertheless, a number of alternative synthetic approaches to synthesize polysilanes have been developed. Among these, the ring opening polymerization of strained cyclosilanes [6,7] and the polymerization of masked disilenes [8] are important, because they permit some control over the stereochemistry of the polymer chain. However, also these methods can not be considered to be general synthetic routes.

Some 20 years ago, Harrod and co-workers discovered the process of dehydrocoupling polymerization of hydrosilanes [9]. They found that group 4 metallocenes, namely dimethyltitanocene and –zirconocene, catalyze silicon-silicon bond formation of phenylsilane accompanied by the loss of hydrogen (Equation 1) [10,11,12,13,14,15,16,17,18,19,20,21].

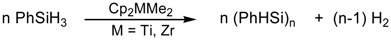
(1)


This reaction promised to be the sought after general synthetic method for the synthesis of polysilanes, which could be used to obtain functionalized polysilanes while also controlling the stereochemistry of the polymer. In the course of investigations concerning transition metal silyl compounds, Tilley and co-workers were able to establish σ-bond metathesis as a major mechanistic pathway of the dehydrocoupling reaction [17,22,23]. Corey *et al.* introduced metallocene dichloride/butyl lithium as an efficient catalyst system [24]. For this and some other systems Harrod and Dioumaev have pointed out a likely involvement of silyl radicals generated via Zr(III)/Zr(IV) redox cycles [23].

Contemporary catalyst systems in olefin polymerization are very similar to the ones used in the silane dehydrocoupling polymerization, and it is well known that polyolefin tacticity can be controlled conveniently by proper choice or design of the catalyst [26]. Therefore, a similar approach to control polysilane tacticity in the dehydrocoupling reaction was tempting. While the main efforts in catalyst design were focused on formation of longer chains and the suppression of cyclic byproducts, two early studies by Harrod [27] and Waymouth [28] reported that some stereocontrol in the polymerization of phenylsilane, employing Brintzinger type (EBTHIZr and EBIZr) catalysts, was achieved [26]. Later, Tanaka’s group reported the synthesis of syndiotactic poly(phenylsilane) employing a (dimethylamino)alkyl substituted CpCp*Zr catalyst system [29].

Nevertheless, Corey and Grimmond later, using Bernoullian statistical analysis of ^29^Si-NMR spectra of polysilane samples, showed that what had been assigned as syndiotactic polysilane is mostly of atactic microstructure [30]. In a related paper, Harrod and coworkers came to essentially the same conclusion, which they derived from their study of the early reaction stages of a polymerization using multidimensional NMR experiments. It was found that at the stage of the tetra- and pentasilanes (the first two chiral oligomers) no stereodifferentiation takes place [31]. This means that *rac* and *meso* isomers are formed to the same extent for the tetrasilane and the same is true for the pentasilane, where the three different possible diastereomers are formed in statistical ratios. If stereocontrol is absent at an early stage of oligomer formation, it can be concluded that any stereodifferentiation present in the molecule is not being caused by the catalyst, which is in line with Corey’s findings [30].

## 2. Results and Discussion

Work from our laboratory on the dehydrocoupling reaction has concentrated mainly on the use of disilanes as starting materials [32,33,34]. Earlier studies by Hengge and Weinberger have shown that the reaction of 1,2-dimethyldisilane catalyzed by dimethylmetallocenes led to a cross-linked, insoluble polymer [35,36]. Comparing this to the good solubility of poly(phenylsilane) it was thought that it would be beneficial to combine the solubility property of poly(phenylsilane) with the cross-linking properties of poly(methyldisilane) in order to obtain a high molecular weight polysilane with good solubility. Therefore, the co-polymerization of 1,2-dimethyldisilane and phenylsilane [37], as well as the polymerization of the single source precursor 1-methyl-2-phenyldisilane (Equations 2 and 3), using Corey’s catalyst system [24] was investigated.

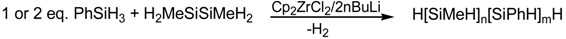
(2)

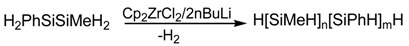
(3)


Molecular weight data of the synthesized polymers are given in Table 1. Interestingly, the molecular weights of the polymers obtained from co-polymerization experiments with varying ratios of PhSiH_3_ and H_2_MeSiSiMeH_2_ were not significantly different from those usually observed in the homo-polymerization reactions of phenylsilane. No cross-linking was detected by GC/MS analysis of the oligomers formed at the beginning of the reaction nor by ^29^Si-NMR spectroscopy. The obtained polymers remained soluble.

**Table 1:** Molecular weights of polysilanes determined by GPC.

Reaction	M_n_	M_w_	D
2 PhSiH_3_ + H_2_MeSiSiMeH_2_	2,100	5,900	2.8
PhSiH_3_ + H_2_MeSiSiMeH_2_	2,500	8,700	3.5
H_2_MeSiSiPhH_2_	3,300	50,000	15.0

**Figure 1:**
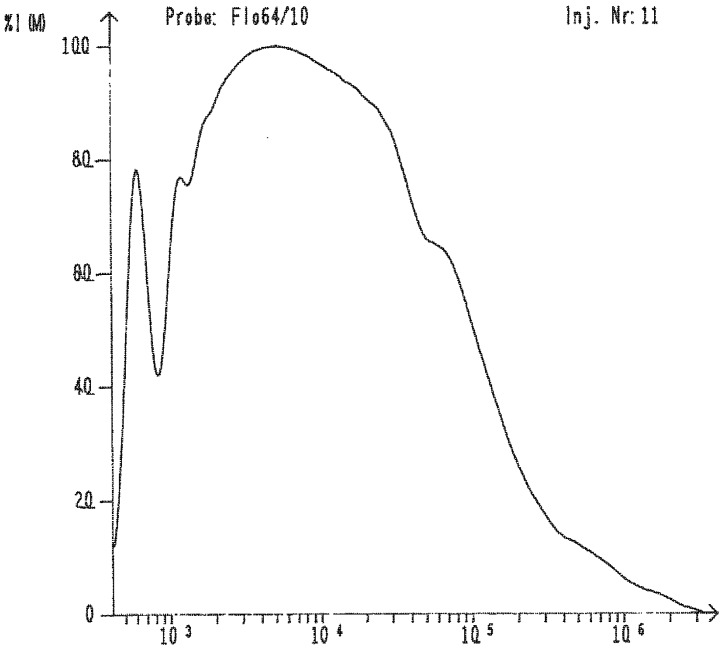
GPC-trace of the polymer obtained from the polymerization of 1-methyl-2-phenyldisilane.

The polymerization of 1-methyl-2-phenyldisilane, however, gave a different result. A polymer with a very broad molecular weight distribution up to 10^6^ Dalton (Table 1, Figure 1) was obtained and a substantial part of the polymer was insoluble in THF and toluene. The insoluble fraction varied from approximately 0−30%. It is likely that this improvement with respect to molecular weight compared to poly(phenylsilane) is due to cross-linking of the methylsubstituted silicon atoms. The unusually high polydispersity index (M_w_/M_n_ = 15) supports this assumption. Also the studies of Hengge and Weinberger showed that in the polymerization of 1,2-dimethyldisilane [36] branched oligomers were formed already at the beginning of the reaction. GPC data of poly(methylsilane), obtained from the reaction of 1,2-dimethyldisilane with dimethylzirconocene, indicated that this polymer becomes insoluble already at a molecular weight of about 10^3^ Dalton, which might be due to the branched/cross-linked structure of the polymer.

In poly(1-methyl-2-phenyldisilane), cross-linking sites seem to be less concentrated than in pure poly(methylsilane) so that most of the polymer remains soluble. Unfortunately, no tertiary Si atoms could be detected by ^29^Si-NMR spectroscopy and the peaks in the ^1^H-NMR spectrum are too broad to provide reliable information to draw any conclusions concerning the concentration of the cross-linking sites.

**Figure 2:**
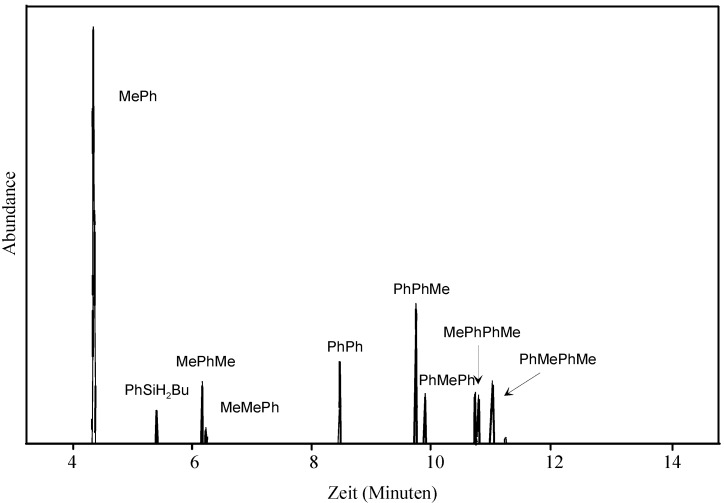
GC/MS analysis of an early stage of the homo-polymerization of 1-methyl-2-phenyldisilane. ^a^

^a^ The designation of peaks to silanes assumes that each substituent (Ph or Me) is attached to and represents one silicon atom in a polysilane chain. All remaining valences are occupied by hydrogen atoms. PhPh therefore refers to 1,2-diphenyldisilane and PhPhMe stands for 1,2-diphenyl-3-methyltrisilane.

GC/MS analysis of the early stages of the polymerization reaction of 1-methyl-2-phenyldisilane led to some interesting insights (Figure 2). Firstly, the disilane is partially cleaved in the polymer build-up reaction since we can observe that the polymer grows by monosilyl units. Thus, the isomers of the direct coupling products of 1-methyl-2-phenyldisilane are not formed to a bigger extent than any other tetrasilane. This result is consistent with the observation of Tilley who also reported the growth of the chain by reaction of a monosilyl unit attached to the metal with a chain of two or more silicon atoms [38].

Secondly, the phenylsilyl unit seems to be considerably more reactive than the methylsilyl group. Among the trisilanes formed, the amount of methyldiphenyltrisilane isomers is substantially higher than that of the dimethylphenyltrisilane isomers. This indicates that the phenylsilyl group is transferred preferentially to the disilane. One possible explanation for this behavior is that methylsilane, which can be formed in the disilane cleavage step, is volatile and thus less abundant in the reaction mixture. Depending on reaction times, the ratio of phenyl to methyl substituted Si-H protons in the ^1^H-NMR analysis of the polymer ranged from 0.9 to 0.65.

A third interesting result was that the isomers in which the “newly attached“ silyl group is coupled to the phenyl-substituted side of the disilane are always formed in a higher number than those where the “newly attached“ silicon atom is connected to the methyl side of the chain. As a consequence, more 1,3-dimethyl-2-phenyltrisilane is formed than 1,2-dimethyl-3-phenyltrisilane [39]. The same is true for 1-methyl-2,3-diphenyltrisilane, which is more abundant than 2-methyl-1,3-diphenyltrisilane. This phenomenon can also be seen in the substitution pattern of the tetrasilanes.

The apparently higher reactivity of the phenyl substituted silicon atoms in the dehydropolymerization reaction could also be observed in the co-polymerization of 1,2-dimethyldisilane with phenylsilane. However, a main difference between the co-polymerization reactions and the homo-polymerization of 1-methyl-2-phenyldisilane is that in the second case a Si-Si bond, which is not so likely to be formed, is already present in the starting material. Although, as stated above, a substantial quantity of starting disilane is cleaved, this still seems to guarantee a higher concentration of methylsilyl units in the polymer and thus also a higher number of potential cross-linking sites. This way, the higher molecular weight of the homo-polymerization reaction can be rationalized.

**Figure 3:**
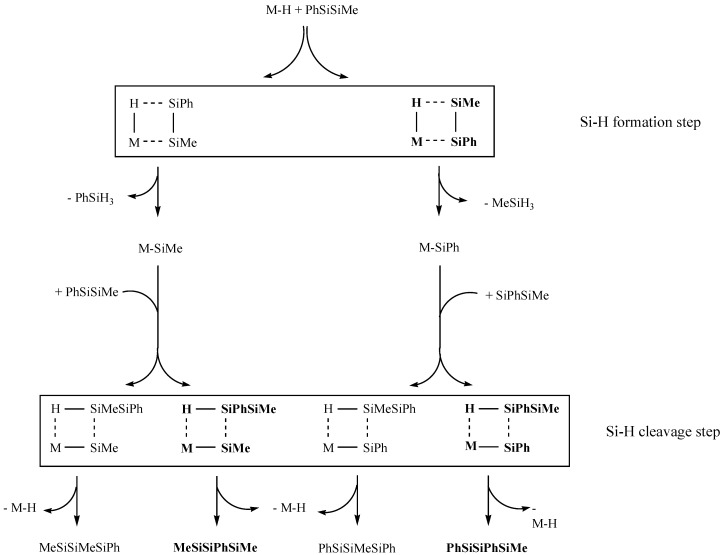
Enhanced reactivity of the phenylsilyl unit compared to the methylsilyl unit. Preferred transition states and isomers (all hydrogen atoms on silicon omitted for clarity) printed bold.

The observed phenylsilyl preference can be explained after a closer look at the two σ-bond metathesis transition states involved in the propagation of the polymer chain (Figure 3). Both include reaction with Si-H bonds; formation in the first step and Si-H bond cleavage in the second step. In each case the silicon atom that carries the involved hydrogen is located in the β-position to the metal. If the thermodynamically more stable Si-H bond is formed and the less stable one is broken preferentially, the Si-H bond strengths of phenylsilane and methylsilane need to be compared. As this bond is slightly stronger in methylsilane than in phenylsilane, the observed pattern makes sense. In the first step the stronger H-SiMe bond is formed, followed by cleavage of the weaker H-SiPh bond in the second step (Figure 3).

**Figure 4:**
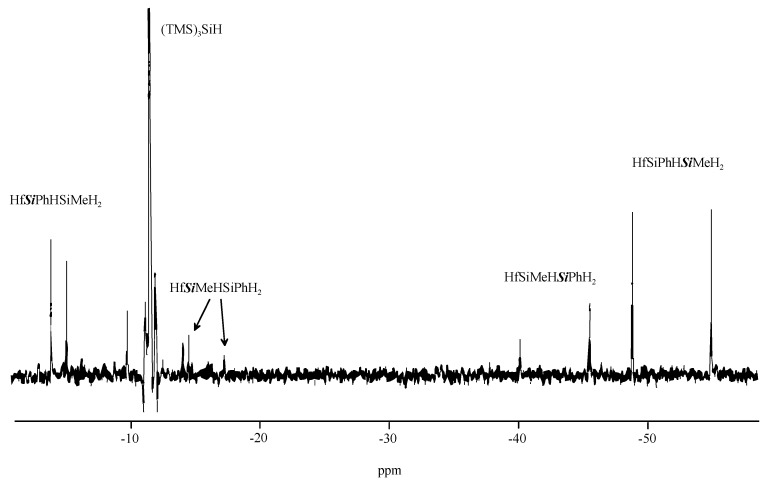
^29^Si{H} INEPT-NMR spectrum of CpCp*Hf(Cl)SiHPhSiMeH_2_/CpCp*Hf(Cl)-SiHMeSiPhH_2_.

To verify this argument the reaction of 1-phenyl-2-methyldisilane with CpCp*Hf(Cl)Si(SiMe_3_)_3_ was investigated. The latter can be used, as was demonstrated by Tilley and co-workers, to obtain stable metal silyl derivatives [40,41] and has already proven to be useful for the elucidation of the σ-bond mechanism [22,23]. The reaction with 1-methyl-2-phenyldisilane should lead to two diastereomeric pairs depending on which end it reacts with the metal (Equation 4). All four compounds were observed by ^1^H and ^29^Si NMR spectroscopy, and it was found that formation of the diasteromeric pair, where the phenyl substituted silicon atom is connected to the metal (1), was about four times higher than the other regioisomers (2) (Figure 4). This is consistent with the argumentation about Si-H bond strength and corresponds approximately to the ratio expected for the 1.4 kcal/mol energy difference tabulated [42]. As expected, the weaker H-SiPh bond is broken preferentially.

Since the obtained complexes feature chiral atoms, these stoichiometric reactions should in principle also allow some conclusions to be drawn on possible stereoselectivity of the first reaction step. The observed diastereomeric ratio of CpCp*(R/S)Hf(Cl)*(R/S)*SiR_3_/CpCp*(R/S)Hf(Cl)*(S/R)*SiR_3_ was exactly 1:1 for both diastereomeric pairs. Further reactions of CpCp*Hf(Cl)Si(SiMe_3_)_3_ with 1,2-dimethyldisilane and 1,2-diphenyldisilane also gave diastereomeric pairs with an exact 1:1 ratio. The same odd non-selectivity was observed before by Tilley [22,23].

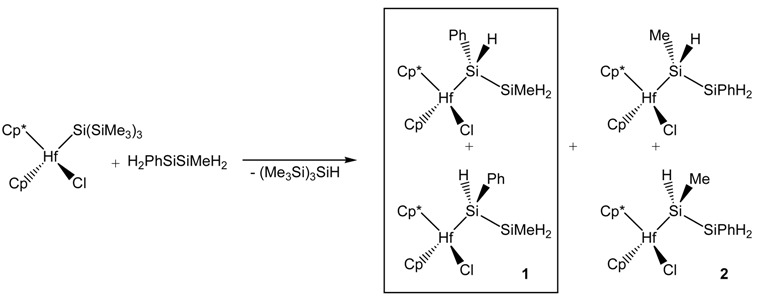
(4)


In regard to the discussion about stereoregulation that is presented in the introduction of this paper, from these examples above, it seems to become obvious that σ-bond metathesis reactions of group 4 metallocene compounds with hydrosilanes display a total lack of stereoselectivity. Thus, the point presented by Harrod *et al.*, which assumes that the lack of stereoselectivity is caused by a mechanism occurring outside the coordination sphere of the metal, is not really required to explain an atactic microstructure.

It is not easy to explain this non-selectivity, but maybe for the polymerization a line of argument that is used in the discussion of hydrogenation and isomerization chemistry can be employed [43]. Tilley has pointed out the similarity of the Ziegler-Natta polymerization as a 2_σ_+2_π_ cycloaddition, to the dehydropolymerization of hydrosilanes, which can be seen as a 2_σ_+2_σ_ process. This is an interesting comparison as it indicates a main difference between the two reactions. In the olefin polymerization, a π-bond adds to the metal-chain bond. This has two important consequences. Firstly, the product of this reaction is another metal-chain compound, the chain remains attached to the metal all the time. Secondly, the reverse reaction is not a likely process. Therefore, whatever stereochemical information has been generated in the addition reaction should not change once it has been established. On the other hand, addition of a σ-bond to a metal-silyl complex results in formation of a compound with a newly formed Si-Si bond. But as a consequence of this, the chain loses contact to the metal, which means that the next interaction of the oligomer with the catalyst can be one of a number of different actions. It can be a chain scission step (*i.e.,* the reverse of the reaction just described) as described for the disilane above. The ease of the reverse reaction has an important implication: it renders any possible kinetic (stereoregulation) effect obsolete and favors a thermodynamic control of the reaction. Alternatively, a chain propagation step can follow, which again can be accomplished in a number of different ways. Since in the formed polysilane all silicon atoms in the chain are chiral, except for the terminal ones, the stereochemical description is more complicated than in the polyolefin case.

## 3. Experimental Section

All manipulations involving air-sensitive materials were performed under a nitrogen or argon atmosphere using standard Schlenk techniques. All solvents were dried over sodium/potassium alloy under nitrogen and distilled prior to use. NMR spectra were recorded on Bruker MSL 300 or DPX 500 spectrometers. Samples were dissolved in C_6_D_6_. Shifts are reported in ppm downfield from TMS (tetramethylsilane). ^29^Si-NMR spectra were obtained using a proton decoupled INEPT pulse sequence [46,47]. GC/MS analyses were carried out on a HP 5890 series II (capillary column DB-1HT; 15 m × 0.251 mm; 0.1 µm) equipped with a with a HP 5971 mass selective detector. GPC analyses were carried out in THF at 25 °C using a Merck-Hitachi L 6200 intelligent pump and columns from POLYMER STANDARDS SERVICE (5 µm, pre-column, mixed gel; 5 µm 10^6^ Å pore diameter, 8 × 300 mm; 5 µm, 10^4^ Å pore diameter, 8 × 300 mm; 5 µm, 10^3^ Å pore diameter, 8 × 300 mm). Polystyrene standards (2.4 × 10^6^ – 665 g/mol) were used for calibration. Methylpentaphenyldisilane [44], 1,2-dimethyldisilane [36], 1,2-diphenyldisilane [45], and CpCp*Hf(Cl)Si(SiMe_3_)_3_ [41] were prepared using established procedures.

### 3.1. Synthesis of 1-methyl-2-phenyldisilane

Trifluoromethanesulfonic acid (17.0 mL, 192.6 mmol) was added drop-wise to a solution of methylpentaphenyldisilane (20.0 g, 43.8 mmol) in benzene (200 mL) at 0 °C. After 96 h at RT, ^29^Si-NMR spectroscopy indicated complete formation of the tetratriflate [δ (ppm): -5.05; ‑26.53]. The solution was cooled in an ice bath and a solution of lithiumaluminumhydride in ether (70.0 mL, 1.5 M solution, 105 mmol) was added drop-wise. After further 2 h at RT, the solution was poured on ice/1 M sulfuric acid. The aqueous layer was extracted twice with ether (2 × 50 mL) and the combined organic layers were dried over sodium sulfate. After distilling off solvents at RT, the residue was distilled (30 Torr/85 °C) to give the product (4.34 g, 67%) as a colorless oil. NMR: ^1^H (300 MHz, C_6_D_6_, r.t.): 7.45 (m, 2H), 7.1 (m, 3H), 4.37 (t, 2H), 3.78 (m, 2H), 0.03 (t, 3H). ^13^C (75.5 MHz, C_6_D_6_, r.t.): 136.57, 130.33, 130.03, 128.89. -10.98. ^29^Si (59.6 MHz, C_6_D_6_, r.t.): -60.25 (^1^J_(SiH)_ = 192.5 Hz), -67.73 (^1^J_(SiH)_ = 187.3 Hz). MS M/z (Int.): 152 (M^+^, 16), 147 (5), 122 (13), 121 (100), 107 (29), 106 (22), 105 (52). IR (cm^-1^): ν−SiH 2135.

### 3.2. General polymerization procedure

Butyllithium (0.34 mL, 1.57 M in hexane, 0.54 mmol) was added drop-wise to a suspension of zirconocenedichloride (80 mg, 0.27 mmol) in toluene (1 mL) at 0 °C. After 30 min a color change to brown was observed.

Polymerization reactions were typically carried out in neat monomer (2 mL) without additional solvent. After addition of the catalyst solution usually a vigorous gas evolution was observed. With increasing viscosity, a strong foaming tendency could be observed sometimes. After 24 h volatiles were removed in vacuum and the residue was dissolved in toluene filtered over a florisil column and eluted with toluene. Addition of hexanes to the solution precipitated the higher polymer fraction, while small cyclic molecules stayed in solution.

### 3.3. Preparation of hafnocene disilanyl compounds

CpCp*Hf(Cl)Si(SiMe_3_)_3_ (50 mg, 0.076 mmol) and the respective disilane (0.076 mmol) were mixed and dissolved in benzene-*d_6_*. Completeness of conversion was detected by ^1^H NMR spectroscopy.

CpCp*Hf(Cl)SiPhHSiMeH_2_ (**1**) *2 diastereomers*: ^1^H NMR (500 MHz, C_6_D_6_, r.t.): 0.31 (m, 6H, β-Si*Me*), 1.76 (s, 15H, Cp*), 1.77 (s, 15H, Cp*), 4.07 (q, 1H, J = 5.4 Hz, β-Si*H*), 4.18 (m, 2H, β-Si*H*), 4.24 (m, 1H, β-Si*H*), 4.46 (dd, 1H, J =1.5, 4.6 Hz, α-Si*H*), 4.56 (d, 1H, J =3.0 Hz, α-Si*H*), 5.71 (s, 5H, *Cp*), 5.72 (s, 5H, *Cp*), 7.08 (m, 2H), 7.21 (m, 4H) 7.68 (d, 2H), 7.77 (d, 2H). ^29^Si NMR (59.6 MHz, C_6_D_6_, r.t.): -55.1 (t, ^1^J_SiH_ = 179 Hz, SiPhH*Si*MeH_2_), -49.0 (d, ^1^J_SiH_ = 179 Hz, SiPhH*Si*MeH_2_), -5.0 (d, ^1^J_SiH_ = 149 Hz, *Si*PhHSiMeH_2_), -3.9 (t, ^1^J_SiH_ = 152 Hz, *Si*PhHSiMeH_2_).

CpCp*Hf(Cl)SiMeHSiPhH_2_ (**2**) *2 diastereomers*: ^1^H NMR (500 MHz, C_6_D_6_, r.t.): 0.48 (d, J = 5 Hz, 3H, α-Si*Me*), 0.69 (d, J = 5.0 Hz, 3H, α-Si*Me*), 1.76 (s, 15H, Cp*), 1.78 (s, 15H, Cp*), 4.21 (m, 1H, α-Si*H*), 4.18 (q, J = 5.5. Hz, 1H, α-Si*H*), 4.60 (s, 1H, β-Si*H*), 4.72 (d, 1H, J =3.1 Hz, β-Si*H*), 4.78 (s, 1H, β-Si*H*), 5.70 (s, 5H, *Cp*), 5.73 (s, 5H, *Cp*), 7.08 (m, 2H), 7.20 (m, 4H) 7.72 (d, 2H), 7.77 (d, 2H). ^29^Si NMR (59.6 MHz, C_6_D_6_, r.t.): -45.7 (SiMeH*Si*PhH_2_), -40.1 (SiMeH*Si*PhH_2_), -17.2 (*Si*MeHSiPhH_2_), -14.5 (*Si*MeHSiPhH_2_).

CpCp*Hf(Cl)SiMeHSiMeH_2_ (**3**) *2 diastereomers*: ^1^H NMR (300 MHz, C_6_D_6_, r.t.): 0.25 (m, 3H, β-Si*Me*), 0.48 (m, 3H, β-Si*Me*), 0.55 (d, J = 4.9 Hz, 3H, α-Si*Me*), 0.80 (d, J = 5.0 Hz, 3H, α-Si*Me*), 1.81 (s, 15H, Cp*), 1.82 (s, 15H, Cp*), 3.95–4.10 (m, 4H, β-Si*H*), 4.15–4.35 (m, 2H, α-Si*H*), 5.84 (s, 5H, *Cp*), 5.87 (s, 5H, *Cp*). ^29^Si NMR (59.6 MHz, C_6_D_6_, r.t.): -54.0 (t, ^1^J_SiH_ = 179 Hz, SiMeH*Si*MeH_2_), -49.3 (t, ^1^J_SiH_ = 166 Hz, SiMeH*Si*MeH_2_), -15.3 (d, ^1^J_SiH_ = 144 Hz, *Si*MeHSiMeH_2_), -12.2 (d, ^1^J_SiH_ = 126 Hz, *Si*MeHSiMeH_2_).

## 4. Conclusions

Comparison between the co-polymerization of phenylsilane and 1,2-dimethyldisilane and the homo-polymerization of the novel single source precursor 1-methly-2-phenyldisilane has provided some interesting insight. Reaction of 1-methly-2-phenyldisilane revealed a preferential reactivity of phenyl substituted silyl units. This is likely caused by the fact that the Si-H bond of a phenyl-substituted silicon atom is weaker than the one on a methyl-substituted silicon atom. Consequently, the disilane interacts preferentially via its phenylsilyl unit with the metal as a strong H-SiMe bond is formed in the generation of methylsilane. The second consequence is that in the Si-Si bond forming step of the catalytic cycle, phenylsilyl units are more likely to occupy the β-position of the four membered transition state as the weaker Si-H bond is broken preferentially.

This mechanistic rationale also explains why the result of the co-polymerization of phenylsilane and 1,2-dimethyldisilane is so similar to a plain polymerization of phenylsilane. The same factors are responsible. In the primary step, mainly phenylsilane adds to the metal as a strong H-H bond is formed. In the secondary step also phenylsilane or a chain with a terminal phenylsilyl unit is involved preferentially. This means that co-polymerization of phenylsilane and 1,2-dimethyldisilane is mainly a polymerization of phenylsilane at least at the beginning of the reaction. Once phenylsilane is consumed, the reaction of 1,2-dimethyldisilane starts and gives as already noted branched insoluble polymer. Of course some co-polymerization does occur, but as the main course of reaction two separate polymerization processes take place. The difference to the homo-polymerization of 1-methly-2-phenyldisilane is therefore caused by the fact that the precursor already contains a bond that is unlikely to be formed in the co-polymerization process. This leads eventually to the presence of a higher number of potential branching points in the polymer.

The phenylsilyl preference in these reactions with the catalyst was found by GC/MS analysis and further substantiated by stoichiometric reaction of 1-methly-2-phenyldisilane with CpCp*Hf(Cl)Si(SiMe_3_)_3_. The main products formed in this reaction are the diastereomers with the phenylsilyl unit attached to the metal. The diastereomeric ratio between the two formed pairs of diastereomers was found to be exactly 1. The same ratio was also found for stoichiometric reactions of 1,2-dimethyldisilane and 1,2-diphenyldisilane with CpCp*Hf(Cl)Si(SiMe_3_)_3_. In summary these results indicate an electronic but certainly no stereo-selectivity in the σ-bond metathesis polymerization of hydrosilanes. The lack of stereoselectivity is consistent with reports on atactic microstructure of polysilanes obtained in dehydrocoupling reactions.
